# Inter-genomic DNA Exchanges and Homeologous Gene Silencing Shaped the Nascent Allopolyploid Coffee Genome (*Coffea arabica* L.)

**DOI:** 10.1534/g3.116.030858

**Published:** 2016-07-19

**Authors:** Philippe Lashermes, Yann Hueber, Marie-Christine Combes, Dany Severac, Alexis Dereeper

**Affiliations:** * UMR DIADE, Institut de Recherche pour le Développement, IRD, Université de Montpellier, 34394 Cedex 5, France; † MGX-Montpellier GenomiX, Institut de Génomique Fonctionnelle, 34094 Cedex 5, France; ‡ UMR IPME, Institut de Recherche pour le Développement, CIRAD, Université de Montpellier, 34394 Cedex 5, France

**Keywords:** polyploidy, evolution, gene conversion, homoeologous recombination, genome dominance

## Abstract

Allopolyploidization is a biological process that has played a major role in plant speciation and evolution. Genomic changes are common consequences of polyploidization, but their dynamics over time are still poorly understood. *Coffea arabica*, a recently formed allotetraploid, was chosen to study genetic changes that accompany allopolyploid formation. Both RNA-seq and DNA-seq data were generated from two genetically distant *C. arabica* accessions. Genomic structural variation was investigated using *C. canephora*, one of its diploid progenitors, as reference genome. The fate of 9047 duplicate homeologous genes was inferred and compared between the accessions. The pattern of SNP density along the reference genome was consistent with the allopolyploid structure. Large genomic duplications or deletions were not detected. Two homeologous copies were retained and expressed in 96% of the genes analyzed. Nevertheless, duplicated genes were found to be affected by various genomic changes leading to homeolog loss or silencing. Genetic and epigenetic changes were evidenced that could have played a major role in the stabilization of the unique ancestral allotetraploid and its subsequent diversification. While the early evolution of *C. arabica* mainly involved homeologous crossover exchanges, the later stage appears to have relied on more gradual evolution involving gene conversion and homeolog silencing.

Polyploidy (the complete doubling of a genome) has long been recognized as an important mechanism in plant speciation and genome evolution (Soltis *et al.* 2014). It is now well established that polyploidy occurred frequently during angiosperm evolution, and that all flowering plant species have undergone one or more rounds of genome duplication in their history (Jiao *et al.* 2011). In particular, allopolyploidization [arising from interspecific hybridization accompanied by whole-genome duplication (WGD)] is considered to have contributed to the adaptation to broader and novel environmental niches, and is thought to have played a fundamental role in the evolutionary history of speciation (Comai 2005; Soltis *et al.* 2014). In addition, many major agricultural crop plants, including wheat (*Triticum aestivum*), cotton (*Gossypium hirsutum*), rapeseed (*Brassica napus*), sugarcane (*Saccharum officinarum*), and coffee (*Coffea arabica*) are allopolyploids.

The establishment of a new allopolyploid species is not a trivial feat. The merging of two or more divergent genomes, and the presence of these parental genomes in duplicate in a single nucleus, can set the stage for dynamic changes to the genome, transcriptome, and phenotype of the new polyploid species (Doyle *et al.* 2008). However, the newly formed allopolyploid faces several immediate challenges. It must secure exclusive intragenomic pairing at meiosis that will lead to full fertility and disomic inheritance. Meiosis can therefore have a dual impact on the evolution of many newly formed polyploids by (1) enabling sexual propagation, and (2) generating, through meiotic errors, large-scale chromosomal variation upon which genetic drift and/or selection can act (Leitch and Leitch 2008). In addition, the newly formed allopolyploid must orchestrate intergenomic interactions and regulate gene expression to adapt to its environment (Jackson and Chen 2010). Hence, successful allopolyploidizations are those that trigger an array of genomic changes that confer evolutionary advantages (Arrigo and Barker 2012).

In the last decade, molecular data from resynthesized and natural allopolyploids has shown that genetic and epigenetic changes are common consequences of polyploidization across a wide range of species (Madlung and Wendel 2013). Nevertheless, little is known about the mechanisms that lead to these changes, and even less about their directed or random nature. In addition, genomic responses to WGD appear to be extremely diverse, and both genomic rearrangements and gene expression changes vary to different degrees depending on the polyploid species concerned, thus preventing simple generalizations. For example, rapid genomic changes have been reported in many new allopolyploids (Doyle *et al.* 2008), but not in allopolyploid cotton (Liu *et al.* 2001). Some polyploid genomes appear to undergo rapid homeolog loss (Gaeta *et al.* 2007; Soltis *et al.* 2010; Buggs *et al.* 2012), whereas, in other polyploids, changes in gene expression appear to dominate (Lee and Chen 2001; Wang *et al.* 2006; Flagel and Wendel 2010; Wang *et al.* 2016). To understand allopolyploid genome evolution in a broad context, genomic data from many more allopolyploids are required.

Among allopolyploid plants, *Coffea arabica* is an interesting case (Lashermes *et al.* 1999, 2000). *C. arabica* is a recent (< 100,000 yr ago) allotetraploid (C^a^E^a^ genome) formed by hybridization between two diploid species: *C. canephora* (C genome) and *C. eugenioides* (E genome). The two parental species are closely related, and the two subgenomes have low sequence divergence (*i.e.*, 1.3% average difference for genes, Cenci *et al.* 2012). In spite of the close relationship between the two constitutive subgenomes, *C. arabica* displays diploid-like meiotic behavior with bivalent formation. In addition, the most recent molecular analyses (Lashermes *et al.* 2014) showed that genomic rearrangements involving homeologous exchanges occurred in *C. arabica*, and could be a major source of genetic diversity. Evidence for a large number of homeologous exchange events (HEEs) shared by all accessions of *C. arabica* strongly supports the hypothesis of a single allopolyploidization event. The highlands of south west Ethiopia are considered as the primary center of genetic diversity of *C. arabica* (Sylvain 1955). The presence of wild populations has been also reported in forest on the Boma Plateau and Mount Imantong of Sudan (Thomas 1942), and in Mount Marsabit of Kenya (Anthony *et al.* 1987). Furthermore, although *C. arabica* accessions exhibit very low genetic diversity (as estimated through molecular markers), they display marked phenotypic/adaptive variation, and there is strong evidence of hybrid vigor when particular accessions are crossed (Van der Vossen *et al.* 2015).

In the present study, *C. arabica* was used to investigate genomic changes in allopolyploid using high-throughput sequencing approaches. Both RNA-seq and DNA-seq data were generated from two genetically distant accessions, and analyzed using *C. canephora* as a reference genome (Denoeud *et al.* 2014). On the one hand, genomic structural variation was investigated based on mapping patterns of DNA sequence reads, and on the detection of copy number alterations (CNA). On the other hand, genes affected by homeolog loss or silencing were inferred by comparing the number of single nucleotide polymorphisms (SNPs) detected in *C. arabica* and between its two diploid progenitor species at both DNA and RNA level. To validate these results, Sanger direct sequencing of cDNA and DNA amplicons from various *C. arabica* accessions was performed. In particular, the distribution of two homeologous crossover exchange events among accessions from the *C. arabica* primary center of diversity was investigated. Genomic rearrangements involving homeologous DNA exchanges, as well as gene conversion and homeolog silencing were evidenced that could have played a major role in the evolution and diversification of *C. arabica*.

## Materials and Methods

### Plant material, library construction, and sequencing

The plant material came from two *C. arabica* accessions, including the commercial cultivar Caturra (an inbred line), one wild accession (AR41) from the ORSTOM (Guillaumet and Hallé 1978) collection mission in Ethiopia, and one accession from each of the two modern-day diploid progenitors, *C. canephora* (acc. DH200-94) and *C. eugenioides* (acc. DA58). Young leaf tissues were collected at the same time from individuals plants grown in a greenhouse in Montpellier (France), and were immediately flash frozen in liquid nitrogen and stored at –80° until DNA and RNA extractions were performed as previously reported (Combes *et al.* 2013; Lashermes *et al.* 2014). For genomic DNA sequencing of the two *C. arabica* accessions, nonindexed paired-ends (PE) libraries were constructed using the TruSeq DNA sample preparation kit (Illumina, San Diego, CA), which includes a fragmentation step by sonication, and, after end-repair and adapter ligation, selection of 470 ± 60 bp DNA fragments by band excision after gel electrophoresis. PE sequencing was carried out at 2 × 100 bp using the Illumina HiSequation 2000, according to the manufacturer’s instructions at the MGX platform (Montpellier Genomix, Montpellier, France). DNA data were collected from two lanes (one lane per library, and per accession) in the same sequencing run. For RNA sequencing of *C. arabica* and *C. eugenioides* accessions, the method and data were as previously reported in Lashermes *et al.* (2014). Briefly, total RNA was isolated from 1 g of material from each plant, and mRNA libraries were constructed using the Illumina “RNA-seq sample prep” kit (Illumina). Single-reads (∼72 nt) were generated with the Illumina HiSequation 2000. For the accession Caturra (*C. arabica*), four independent libraries were constructed from four different plants. The entire sequence dataset has been deposited with the European Nucleotide Archive under the study accession numbers PRJEB5543 and PRJEB9368 for RNA-seq and DNA-seq, respectively.

### Analysis of DNA-seq data

Sequences were first cleaned to remove adapter sequences and quality filtered (phred score > 28). After trimming, reads of < 50 bp were discarded. PE sequences were then mapped onto the total *C. canephora* (acc. DH200-94) reference genome (http://coffee-genome.org, Denoeud *et al.* 2014) using the BWA-MEM algorithm (Li and Durbin 2009; http://bio-bwa.sourceforge.net/) with the PE mode and default parameters. The resulting BAM files of unambiguously aligned sequences were then analyzed for SNP discovery with the GATK toolkit (http://www.broadinstitute.org/gatk/), using the Unified-Genotyper module with default parameters. For the detection of copy number changes, the BAM files were analyzed with FREEC (control-Free Copy number caller, Boeva *et al.* 2011) using a nonoverlapping 50-kb sliding window. The main steps are (1) normalization of the copy number profiles using GC content, (2) segmentation of normalized profiles, and (3) assignment of copy number changes to losses and gains.

SNP density was estimated along the 11 homeologous chromosome groups, and regions exhibiting homeologous SNP deficit (HSD) were identified using in-house Perl-scripts (available upon request). Nonoverlapping 10-kb sliding windows were used to estimate the SNP density along the *C. canephora* reference genome. To minimize the rate of false-positive SNPs, a minimum depth coverage of 10 was required for a position to be considered, while positions exhibiting depth coverage greater than twice the overall sample mean were discarded. Since the genome sequence of *C. eugenioides* is not yet available, expected local SNP density (in the absence of genetic changes in *C. arabica*) along the homeologous chromosome groups could not be determined. So, the overall mean SNP density was used as reference value, and the hypothesis of HSD was retained when at least five consecutive 10-kb windows (presenting a minimum of 8000 positions that satisfied the depth criteria) along the *C. canephora* reference genome were identified as exhibiting a highly significant SNP deficit using a test of proportions with R software (prop.test; Newcombe 1998). To control for multiple testing, the resulting P values were corrected using the Benjamini and Hochberg (1995) procedure. A cumulative window size of 50 kb was found to produce a good compromise between informativeness and accuracy.

### RNA-seq data processing

The 72-nt reads of each library were mapped to a *C. canephora* coding DNA sequence reference (25574 CDS) as transcriptome reference using BWA MEM with the default parameters (http://coffee-genome.org; Denoeud *et al.* 2014). The aligned sequences of each library were then analyzed to find SNPs with the GATK toolkit using the Unified-Genotyper module with default parameters to obtain a list of SNPs and allelic data, and the Depth-Of-Coverage module to obtain information on depth coverage. Regarding the accession Caturra, analyses were performed using either a pool of reads from the four libraries or each individual library. To avoid artifacts due to reads from pseudogenes or repeat sequences, only CDS identified as single copy were used for subsequent analyses.

### Comparison of SNPs determined by RNA-seq and DNA-seq

SNPs were quantified and compared using SNiPlay (http://www.southgreen.fr/)—a dedicated web-based tool (Dereeper *et al.* 2011). In particular, SNiPlay authorizes the user to set minimum depth coverage for a sequence position to be taken into consideration. To allow combined analysis of SNP-gene data generated by both RNA-seq and DNA-seq, the list of SNPs, allelic data, and depth coverage corresponding to the 25574 CDS reference were extracted from the whole genome DNA-seq data using the in-house Perl-script (available upon request) and the GFF file of the *C. canephora* reference genome (http://coffee-genome.org). For each gene, the SNPs detected in each of accessions analyzed by either DNA-seq or RNA-seq were determined and common sequence positions were compared. A minimum depth coverage of 12 was required for the DNA-seq in *C. arabica* to ensure good coverage of the two subgenomes, while the RNA-seq read cutoff was set at four and 16 for *C. eugenioides* and *C. arabica*, respectively, to account for the possibility of SNP allele low frequency due to homeolog expression bias in the allopolyploid (Yoo *et al.* 2014; Combes *et al.* 2013). In addition, SNP cut-off parameters were applied throughout the analyses in order to take into account the occurrence of sequencing (or SNP detection) errors, and the fact that the two diploid parental accessions used in this experiment are not the true diploid progenitors of the allotetraploid *C. arabica*.

**Figure 1:**
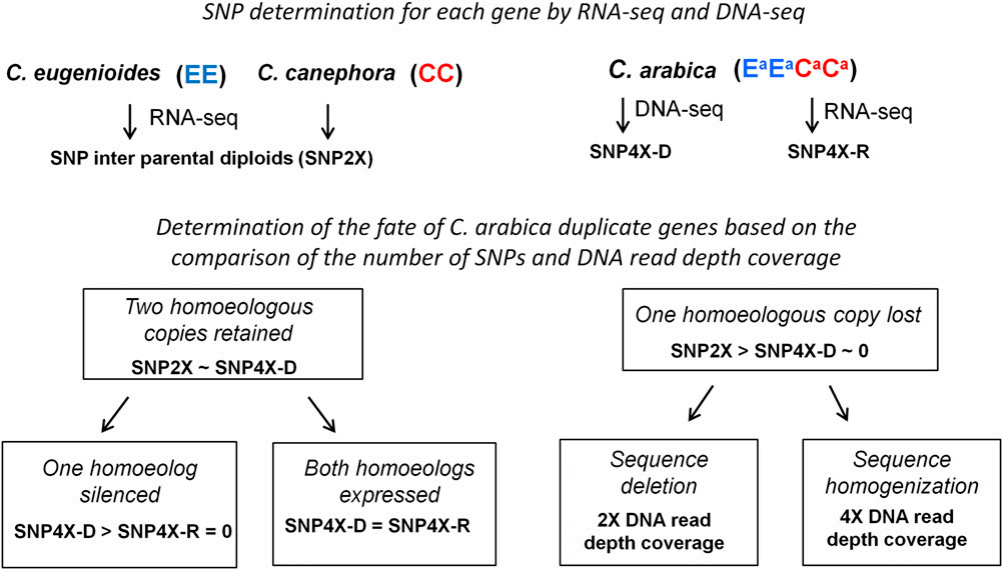
Flow chart of methods used to analyze the fate of duplicate genes in allotetraploid *C. arabica*.

The fate of homeologous genes in *C. arabica* was investigated as shown in Figure 1. For each gene, SNP number and DNA read depth coverage comparisons were carried out. A minimum of three SNPs between the two diploid species, as well as in the allopolyploid, as determined by DNA-seq were selected as the cutoff to set a lower bound on the resolution of SNP number change for a given gene. First, the number of SNPs determined by DNA-seq in the allopolyploid (SNP4X-D) was compared to the number of SNPs between the two diploid species (SNP2X). For a given gene, when SNP2X and SNP4X-D were comparable, two homeologous copies were considered to have been retained. In contrast, genes displaying at least a fourfold decrease between SNP2X and SNP4X-D, and no more than one SNP classified as homeologous (SNP4X-D ≤ 1) were considered as exhibiting homeolog loss in the *C. arabica* accession concerned (when SNP4X-D was null, a minimum value of three was required for SNP2X). Second, for each gene presenting two homeologous copies, the number of SNPs determined in the allopolyploid by RNA-seq (SNP4X-R) and DNA-seq (SNP4X-D) were compared. Genes in the allopolyploid displaying at least three SNPs at the DNA level (SNP4X-D ≥ 3) and no SNPs at the RNA level (SNP4X-R = 0; presence of one SNP was tolerated if different than those detected by DNA-seq) were considered as exhibiting homeolog silencing in the *C. arabica* accession concerned. Others genes displaying similar SNPs at the DNA and RNA levels were considered as expressing both homeologs. Finally, for each gene presenting one homeolog loss, the average read coverage was compared to the expected values (99% confidence interval) for a gene with two copies and four copies, respectively, as reported previously (Lashermes *et al.* 2014). According to the gene read depth coverage categorization, the homeolog loss was inferred to result from either sequence deletion (*i.e.*, two copies) or sequence homogenization (*i.e.*, four copies).

### Analysis of genes exhibiting homeolog loss and homeolog silencing

In each gene involved in homeolog loss and homeolog silencing, the numbers of SNPs shared by *C. arabica* and either *C. canephora* or *C. eugenioides* were compared to identify the subgenome in which the event occurred. Homeolog loss/silencing was attributed to the subgenome deriving from the diploid progenitor species with the smallest number of SNPs shared with *C. arabica*. A difference between the two SNP categories of at least three SNPs was required to determine the subgenome in which the homeolog loss or silencing occurred.

Computational mapping and Plant GO-slim annotation were performed using Blast2GO software v2.6.4 (http://www.blast2go.org; Conesa and Gotz 2008) as described in Combes *et al.* (2013). Functional enrichments in groups of interesting genes were investigated using Fisher’s exact test, applying a false discovery rate (FDR), and a correction for multiple testing.

### Validation of genes exhibiting homeolog loss and silencing

Experimental validation of bioinformatically inferred genes exhibiting either homeolog lost or silencing was performed using traditional Sanger sequencing, as previously reported in Combes *et al.* (2012). For homeolog loss analysis, three genes from the two main regions (*i.e.*, B and C, see Table 2) showing contiguous genes exhibiting homeolog losses in both accessions analyzed were selected (Supplemental Material, Table S4). Primer pairs were designed to amplify DNA fragments containing species-specific SNPs that differentiate the two diploid progenitor species, *C. canephora* and *C. eugenioides* (Combes *et al.* 2015). SNP detection assays were performed based on Sanger direct sequencing of DNA amplicons from 96 accessions originating from the main regions of *C. arabica* primary center of diversity (Labouisse *et al.* 2008) (Table S5). For silencing validation, a subset of five genes was selected, and primer pairs were designed to amplify single-exon fragments containing several homeologous SNP (Table S4). The expression of homeologs was analyzed using a SNP ratio quantification method based on dideoxy-terminated sequences of cDNA and DNA amplicons from *C. arabica* (acc. Caturra, AR41 and AR59) as described in Combes *et al.* (2012). PCR reactions were performed twice in a volume of 25 µl, with either 1 µl of the diluted (one-tenth) cDNA generated by the first-strand synthesis or 25 ng of genomic DNA, 0.4 µM of each primer, 2.5 mM MgCl_2_, and 1 U of *Taq* DNA Polymerase. Cycling was done in a GeneAmp PCR 9700 thermocycler for 2 min at 94° followed by five cycles of 10 sec at 94°, 30 sec at 60° to 55° (–1°/cycle), and by 30 cycles of 10 sec at 94°, 30 sec at 55°, 30 sec at 72°, and a final 8-min extension at 72°. Sequencing was performed by Beckman Coulter Genomics (Takeley UK). Sequence chromatograms of exon portions amplified on cDNA and DNA were analyzed and compared using BioEdit (Hall 1999).

### Data availability

The authors state that all data necessary for confirming the conclusions presented in the article are represented fully within the article. The entire sequence dataset has been deposited at European Nucleotide Archive under the study accession numbers PRJEB5543 and PRJEB9368 for RNA-seq and DNA-seq, respectively.

## Results

### Genomic structural variation

Genome sequencing of the two genetically distant accessions of *C. arabica* yielded 286 million 100-bp PE reads comprising 57.3 Gb of raw data (Table S1). After cleaning, 267 million (*i.e.*, 93%) of the reads remained, and were mapped to the high-quality draft *C. canephora* genome sequence. The number of reads mapped onto the genome ranged from 106 million in accession AR41 to 159 million in accession Caturra. The average sequence depth of coverage of the reference canephora genome varied from 36× to 51× depending on the *C. arabica* accession.

**Figure 2:**
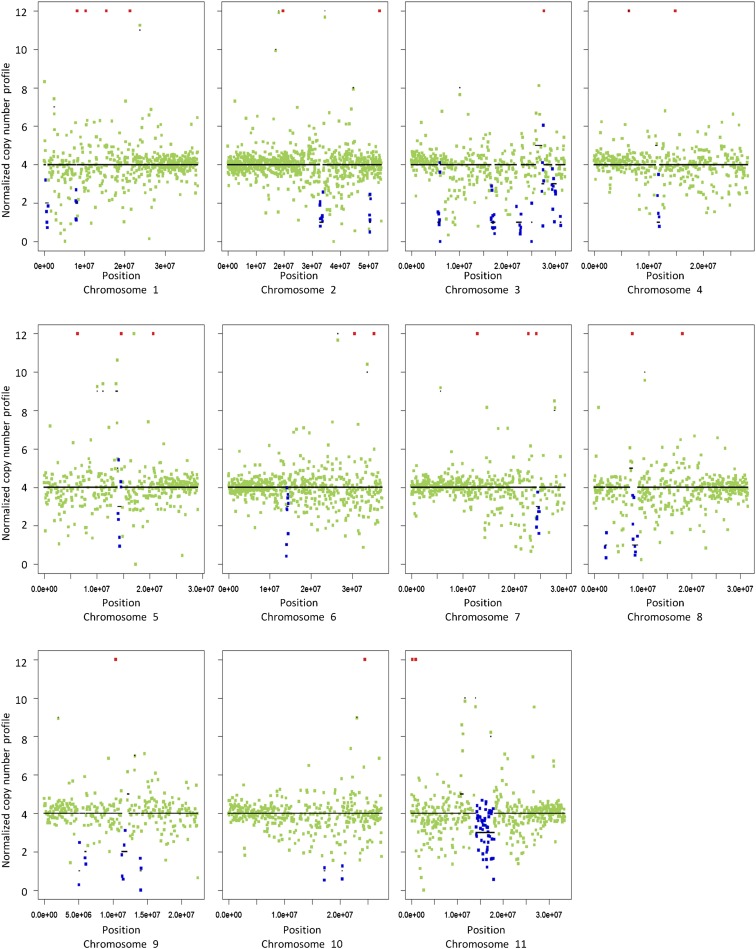
GC-content normalized DNA copy number profile, and FREEC-predicted copy number alteration (Red: gains; blue: losses) for the *C. arabica* genome (acc. Caturra) using a nonoverlapping 50-kb sliding window and the 11 chromosomes of *C. canephora* as genomic reference sequence. Automatically predicted copy numbers are shown in black (line).

CNA based on the depth of coverage was assessed using FREEC (Boeva *et al.* 2011). Copy number profiles were established for the two *C. arabica* accessions using a nonoverlapping 50-kb sliding window, and normalization of GC content. The outcome for the accession Caturra is shown in Figure 2 as an example. Although few CNAs were predicted for each accession, the overall profiles appeared to be consistent with a 4× copy number along the 11 *C. canephora* chromosomes used as reference. Gross copy number changes due to large genomic duplications or deletions were not detected. These results are coherent with the expected allotetraploid structure of *C. arabica*.

**Figure 3:**
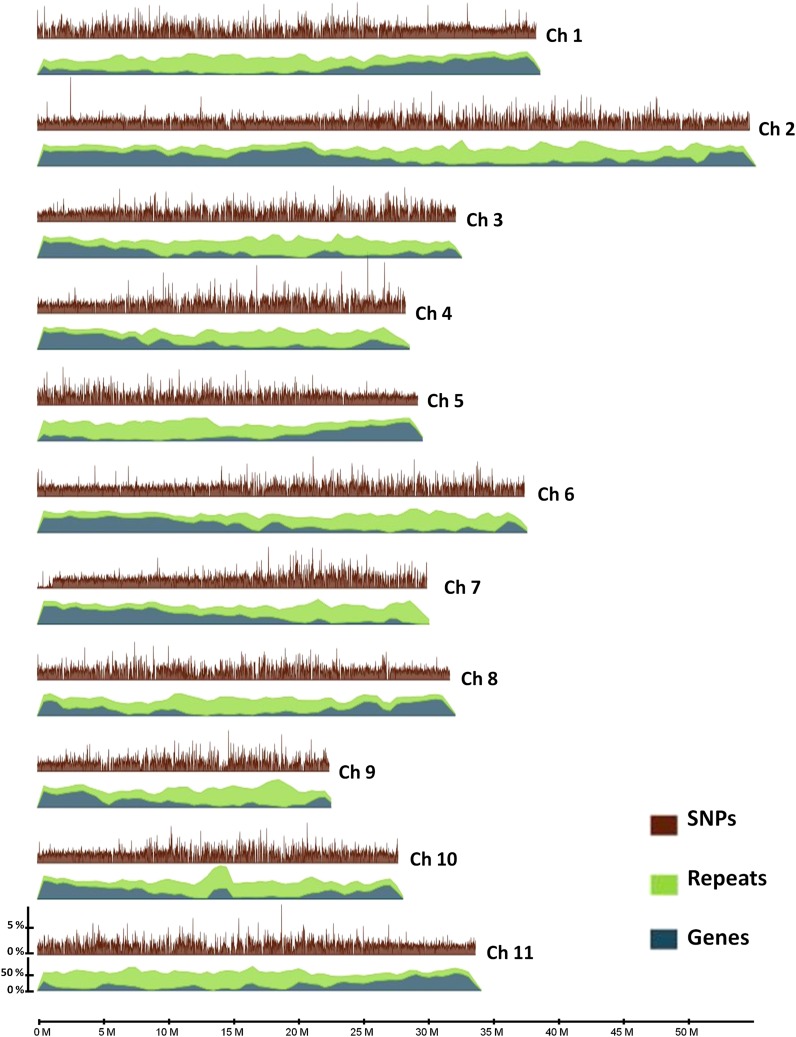
SNP density along the 11 homeologous chromosome groups in *C. arabica* (acc. Caturra). The 11 chromosomes of *C. canephora* (acc. DH200-94) were used as reference genome. Nonoverlapping 10-kb sliding windows, and coverage criteria required to consider a position to minimize the rate of false SNPs, were applied to estimate the density of SNPs in *C. arabica*. The relative proportion (percentage nucleotides) in *C. canephora* (1-Mb sliding window) of transposable elements (green) and genes (blue) are shown at the bottom.

**Figure 4:**
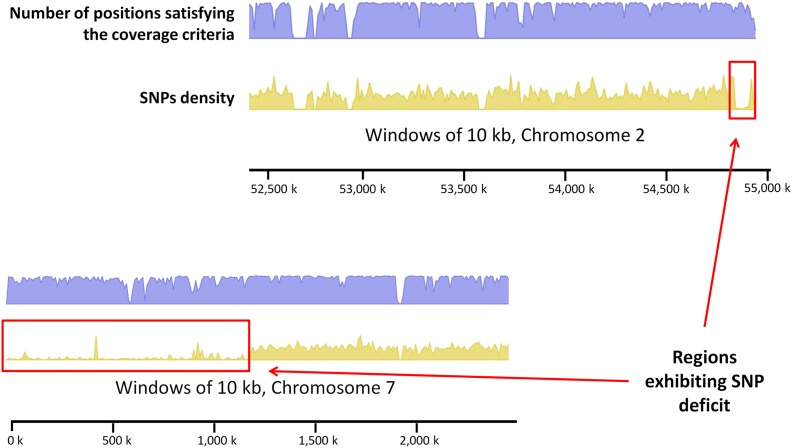
Examples of regions exhibiting homeologous SNP deficit in *C. arabica* (acc. Caturra) on homeologous chromosome groups 2 and 7. Nonoverlapping 10-kb sliding windows were used to estimate SNP density in *C. arabica* (Yellow track). To minimize the rate of false-positive SNPs, a minimum depth coverage of 10 was required for a position to be taken into consideration, and positions with a depth coverage more than twice the overall sample mean were discarded (Blue track).

SNPs were detected in each of the two accessions of *C. arabica*. In allopolyploids such as *C. arabica*, sequence variations between subgenomes (homeologous SNPs) coexist with allelic variations within subgenomes (homologous SNPs). However, given the high level of homozygosity in *C. arabica* (Lashermes *et al.* 2014), it was assumed that most of the identified SNPs are homeologous SNPs. Nonoverlapping 10-kb sliding windows and coverage criteria required to consider a position to minimize the rate of false SNPs were applied to estimate the density of SNPs along the 11 homeologous chromosome groups. Whatever the accession considered, the overall SNP density appeared to be relatively constant along the different reference chromosomes, with a slight increase in the genomic regions enriched in transposable elements (Figure 3). In the two accessions and the regions analyzed, the structural heterozygosity observed was in line with what is expected in an allotetraploid. Nevertheless, regions exhibiting homeologous SNP deficit (HSD) were identified (Figure 4), *e.g.*, 39 regions exhibiting HSD were revealed in *C. arabica* acc. Caturra (Table S2). Of these regions, 37 (*i.e.*, 95%) were shared by the two accessions analyzed. In addition, except the region (1170 kb in size) identified on chromosome 7, the regions were rather small, ranging from 50 kb to 160 kb with an average of 81 kb. The overall size of these regions was 4230 kb, representing 1.5% of the genome windows analyzed that satisfied the depth criteria.

### Evidence of homeolog loss

The fate of homeologous genes in *C. arabica* was inferred as described in Figure 1. The number of SNPs per gene detected by DNA-seq in individual accessions of *C. arabica* were compared to those detected between accessions of its two diploid progenitor species in common sequence positions. A set of 9047 genes that satisfied depth and quality requirements was analyzed in the two arabica accessions. While two homeologous copies appeared to be retained in 98% of the genes, 179 genes (2.0%) exhibiting homeolog loss were identified (Table 1). Although the number of homeolog losses varied from 148 (Caturra) to 174 (AR41) among the two *C. arabica* accessions, a large proportion (80%) of genes exhibiting homeolog loss was shared by the two accessions.

**Table 1:** Determination of the subgenome origin of homeolog loss (A) and silencing events (B) detected in two accessions of *C. arabica*

**(A)** ***C. arabica* Accession**	**Number of Homeolog Loss Events**	**Subgenome Origin of Homeolog Losses**		
		**C^a^**	**E^a^**	**Not Determined**
AR41	174	120	39	15
Caturra	148	110	27	11
Loss shared by both accessions	143	108	25	10
Loss not shared	36	14	17	5

**(B)** ***C. arabica* Accession**	**Number of Silenced Genes**	**Subgenome Origin Of Silenced Genes**		
		**C^a^**	**E^a^**	**Not Determined**
AR41	120	61	37	22
Caturra	116	60	39	17
Silencing shared by both accessions	80	47	24	9
Silencing not shared	76	30	33	13

**Figure 5:**
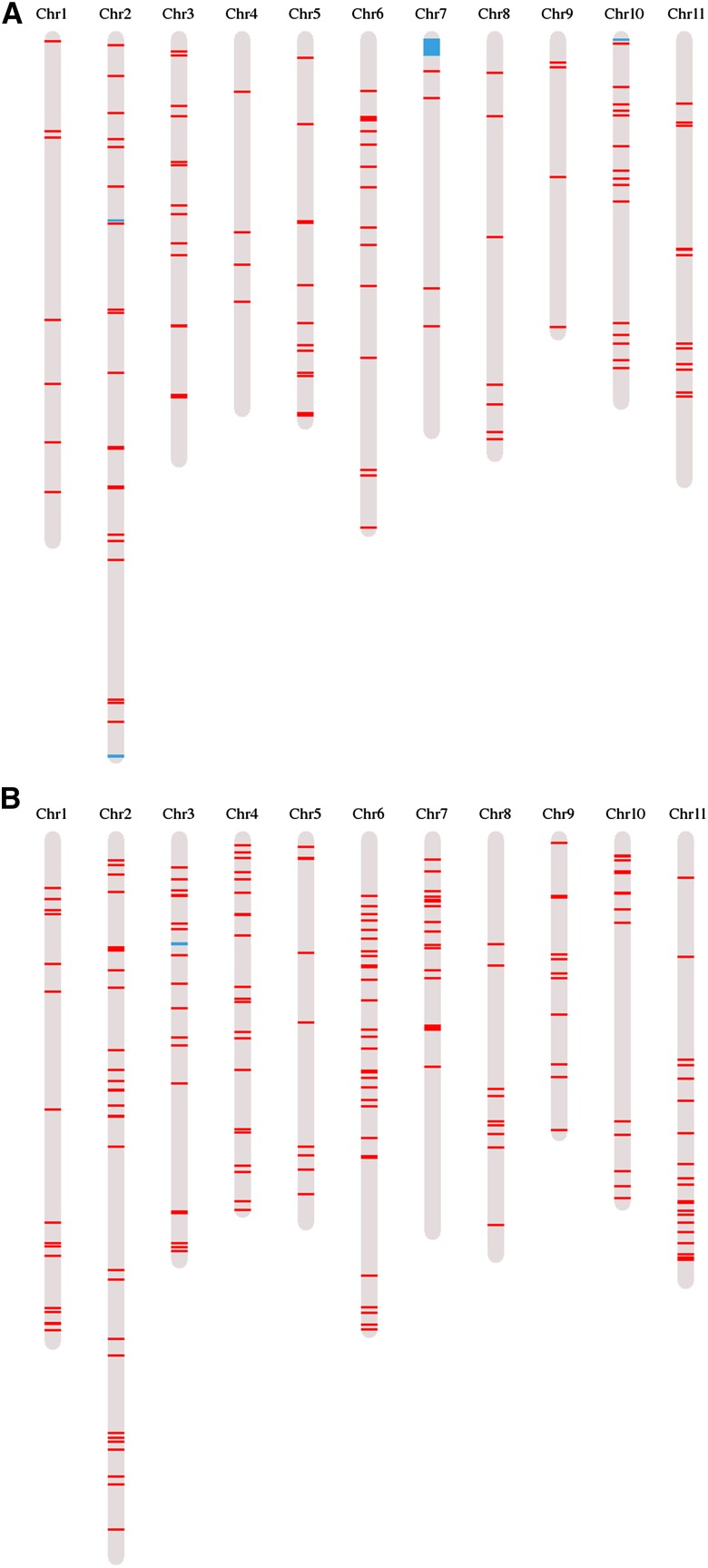
Distribution of loci exhibiting either homeolog loss (A) or homeolog silencing (B) identified in *C. arabica* (acc. Caturra) across *C. canephora* reference chromosomes. Single events of either homeolog loss or homeolog silencing are in red, and regions carrying contiguous genes exhibiting either homeolog loss or homeolog silencing are in blue.

The distribution across the reference genome of loci exhibiting homeolog loss was next investigated (Figure 5). While homeolog loss events were observed across the 11 reference chromosomes, four regions carrying contiguous genes exhibiting homeolog losses were detected (Table 2). The number of genes per region in the accession Caturra ranged from three to 142, and the corresponding genome fragment size ranged from 15 kb to 1198 kb. The four regions were shared by the two accessions, and corresponded to genomic regions previously identified as exhibiting HSD. In contrast, all the single genes exhibiting homeolog losses that did not belong to the four identified clusters corresponded to genome regions (10 kb windows) exhibiting standard SNP density.

**Table 2:** Characterization of four regions in *C. arabica* (acc. Caturra) carrying contiguous genes exhibiting homeolog losses, using the 11 chromosomes of *C. canephora* as genomic reference sequence

**Region Code**	**Homeologous Chromosome Groups**	**Number of Genes Exhibiting Homeolog Loss**	**Gene Identifier (Start/End)**	**Size (kb)**
A	2	4	Cc02g15410/Cc02g15440	15.4
B	2	7	Cc02g39930/Cc02g39990	68.0
C	7	142	Cc07g00010/Cc07g01770	1197.9
D	10	3	Cc10g00010/Cc10g00030	27.8

The subgenome origin of the detected homeolog loss events was further investigated (Table 1). Homeolog loss was attributed to the subgenome deriving from the diploid progenitor species with the least SNPs shared with *C. arabica*. Whatever the accession, homeolog losses were attributed to the two subgenomes, but with a marked preference (at least 75%) for subgenome C^a^. Contrasted behavior was observed between the groups of genes exhibiting homeolog loss shared by the two accessions, or restricted to one accession. While the homeolog losses shared by accessions were attributed mainly to subgenome C^a^ (*i.e.*, 81%), the subgenome origin of homeolog loss events specific to one accession appeared to be balanced between C^a^ and E^a^.

The mechanisms behind the homeolog loss events were further investigated. To distinguish between sequence deletion and sequence homogenization, the gene copy numbers in *C. arabica* were estimated using the DNA-seq read depth of coverage (Figure S1). Indeed, homeolog sequence deletion in an allotetraploid is expected to be associated with a decrease in gene copy number from 4× to 2×. The distribution of genes exhibiting homeolog loss according to their average read depth was very similar to the distribution of the overall genes, supporting the hypothesis that most of the observed homeolog losses are not associated with mere gene loss. Nevertheless, genes that exhibited an average read depth corresponding to the expected coverage for gene in two copies were overrepresented in the group of genes exhibiting homeolog loss (Chi-squared test, P-value < 0.0001) suggesting that a few homeolog loss events could result from sequence deletion. For instance in *C. arabica* acc. Caturra, of the 148 genes identified as exhibiting homeolog loss, 12 (8.1%) displayed an average read depth corresponding to the expected coverage for gene in two copies.

Homeolog loss was validated using direct Sanger sequencing of amplicons for three genes (Table S4) from the two main regions showing contiguous genes exhibiting homeolog losses in the two examined arabica accessions (*i.e.*, regions B and C, see Table 2). The frequency of these homeolog loss events among the *C. arabica* germplasm was further investigated. These events appeared shared by all of the 96 analyzed accessions that represent the main coffee growing regions in the *C. arabica* primary center of diversity.

### Evidence of homeolog silencing

The occurrence of homeolog silencing in *C. arabica* accessions was inferred by comparing the number of SNPs determined by DNA-seq (SNP4X-D) and RNA-seq (SNP4X-R) in common sequence positions in each individual gene (Figure 1). It was assumed that, in the absence of homeolog silencing in *C. arabica*, the number of SNPs determined by DNA-seq and RNA-seq would be equivalent, whereas the failure to detect homeologous SNPs by RNA-seq would reveal homeolog silencing. For most of the 9047 genes analyzed in the two accessions, the number of SNPs determined by DNA-seq and RNA-seq was comparable, and, overall, 95% of the SNPs identified with the two methods were similar. Taking into account the two *C. arabica* accessions, 156 genes (1.7%) exhibiting homeolog silencing were identified (Table 1). The number of genes exhibiting homeolog silencing varied from 116 (Caturra) to 120 (AR41) between the two accessions of *C. arabica*, with 80 genes (51.3%) shared by the two accessions, and 49% of the silenced genes specific to one of the accessions. Homeolog silencing were examined among the four individual plants of the accession Caturra. After filtering for the depth coverage, 66 of 120 genes exhibiting homeolog silencing in Caturra were investigated. For all of them, homeolog silencing was observed in all individual plants.

The distribution across the reference genome of loci exhibiting homeolog silencing was next investigated. The distribution observed for accession Caturra is shown in Figure 5 as an example. Homeolog silencing events were observed across the 11 reference chromosomes, and two small regions (92 kb and 59 kb respectively on chromosome 2 and unanchored scaffold) carrying a cluster of genes exhibiting homeolog silencing were detected in two accessions.

The subgenome origin of the homeolog silencing events was further investigated (Table 1). Homeolog silencing was attributed to the subgenome deriving from the diploid progenitor species displaying the smallest number of SNPs shared with *C. arabica*. Whatever the accession, homeolog silencing was attributed to the two subgenomes with a marked preference for subgenome C^a^ (from 60.6% to 62.2% depending on the accession). This preference (*i.e.*, 66.2% of C^a^ silenced homeologs) was observed particularly among the group of genes exhibiting homeolog silencing shared by the two accessions.

Homeolog silencing was validated using direct Sanger sequencing of cDNA and DNA amplicons from *C. arabica* (acc. Caturra, AR41 and AR59). Primer pairs were designed to amplify single-exon fragments of five genes for which homeolog silencing was bioinformatically inferred (Table S2). For all the genes assayed, expression of only one homeolog from either the subgenomes C^a^ or E^a^ was detected as expected from the previous analyses.

### Gene ontology enrichment analysis

Gene ontology (GO) enrichment was analyzed in groups of genes showing either homeolog loss or homeolog silencing using the full set of analyzed genes as reference group. No significant variation was observed in the group of genes exhibiting homeolog loss, whereas statistically highly significant impoverishments in very general GO terms were observed in the group of genes exhibiting homeolog silencing (Table S3). In particular, specific functions related to macromolecular biosynthesis and organic cyclic compound metabolism were under-represented in the group of genes exhibiting homeolog silencing shared by the two accessions (Table 3).

**Table 3:** Gene ontology enrichment analysis of genes exhibiting homeolog silencing shared by the two accessions of *C. arabica* analyzed

**GO-ID**	**Term**	**Category**	**FDR**	**P-Value**	**Test**	**Ref**	**notAnnotTest**	**notAnnotRef**	**Over/Under**
GO:0043229	Intracellular organelle	C	4.151711E-5	3.610184E-7	13	3977	53	3898	Under
GO:0043226	Organelle	C	4.151711E-5	3.610184E-7	13	3977	53	3898	Under
GO:0043227	Membrane-bounded organelle	C	7.544177E-5	1.312031E-6	13	3881	53	3994	Under
GO:0043231	Intracellular membrane-bounded organelle	C	7.544177E-5	1.312031E-6	13	3881	53	3994	Under
GO:0005623	Cell	C	1.361508E-4	3.219604E-6	24	5116	42	2759	Under
GO:0044464	Cell part	C	1.361508E-4	3.561238E-6	24	5098	42	2777	Under
GO:0044424	Intracellular part	C	1.361508E-4	4.143721E-6	19	4510	47	3365	Under
GO:0034641	Cellular nitrogen compound metabolic process	P	1.882623E-4	6.944386E-6	2	1852	64	6023	Under
GO:0005622	Intracellular	C	1.882623E-4	7.366786E-6	21	4679	45	3196	Under
GO:0006725	Cellular aromatic compound metabolic process	P	5.411463E-4	3.058653E-5	2	1720	64	6155	Under
GO:1901360	Organic cyclic compound metabolic process	P	5.411463E-4	3.058653E-5	2	1720	64	6155	Under
GO:0046483	Heterocycle metabolic process	P	5.411463E-4	3.058653E-5	2	1720	64	6155	Under
GO:0006139	Nucleobase-containing compound metabolic p.	P	5.411463E-4	3.058653E-5	2	1720	64	6155	Under
GO:0005634	Nucleus	C	8.456478E-3	5.147422E-4	2	1413	64	6462	Under
GO:0044444	Cytoplasmic part	C	8.769347E-3	6.260661E-4	14	3281	52	4594	Under
GO:0010467	Gene expression	P	8.769347E-3	6.331017E-4	0	912	66	6963	Under
GO:0003676	Nucleic acid binding	F	8.769347E-3	6.481691E-4	1	1146	65	6729	Under
GO:0034645	Cellular macromolecule biosynthetic process	P	1.039839E-2	9.925819E-4	0	860	66	7015	Under
GO:0044249	Cellular biosynthetic process	P	1.039839E-2	9.925819E-4	0	860	66	7015	Under
GO:0009059	Macromolecule biosynthetic process	P	1.039839E-2	9.925819E-4	0	860	66	7015	Under
GO:0044271	Cellular nitrogen compound biosynthetic proc.	P	1.039839E-2	9.925819E-4	0	860	66	7015	Under
GO:1901576	Organic substance biosynthetic process	P	1.039839E-2	9.946287E-4	0	861	66	7014	Under
GO:0006807	Nitrogen compound metabolic process	P	1.267849E-2	1.267849E-3	7	2175	59	5700	Under
GO:0005737	Cytoplasm	C	2.685331E-2	2.802085E-3	19	3719	47	4156	Under
GO:0044237	Cellular metabolic process	P	2.979136E-2	3.238192E-3	14	3065	52	4810	Under

Analysis was performed using the full set of 9047 analyzed genes as reference group and Fisher’s exact test with a false discovery rate (FDR) correction for multiple testing.

## Discussion

Plant allopolyploidy appeared to be associated with an array of rapid genomic changes in genetic/epigenetic, transcriptomic, and proteomic layers that may affect the fitness of the newly formed allopolyploid and increase its competitiveness, leading to its successful establishment in nature (Arrigo and Barker 2012). In the present study, several approaches based on high-throughput sequencing technologies were used to investigate these genetic changes in *C. arabica*—a model allopolyploid perennial plant. The use of a high-quality draft genome sequence of *C. canephora* (Denoeud *et al.* 2014) as genome and transcriptome references offered new opportunities for analyses compared with previous works (Lashermes *et al.* 2014). However, inherent limitations remained such as the impossibility to study putative genomic regions of *C. arabica* that do not have a counterpart in the *C. canephora* reference genome, and the fact that interpretation of the subgenome (*i.e.*, parental) of the identified homeologous SNPs was limited to the part of the genome for which reference sequences of both diploid parents were available. Nevertheless, whole genome analyses and comparison of two geographically and genetically distant accessions of *C. arabica* provided compelling evidence for the efficiency of sequencing based methods for the investigation of genetic changes in allopolyploid, and of the mechanisms that lead to these changes, their timing, and their directed or random nature.

### Fixed heterozygosity and allopolyploid structure

Although challenging because of the low divergence between the two diploid constitutive subgenomes, the allotetraploid genome organization of *C. arabica* was first revealed by genomic *in situ* hybridization (Lashermes *et al.* 1999). The present data confirm a state of fixed heterozygosity related to the presence of two complete sets of homeologous chromosomes in *C. arabica*. Whatever the accession considered, the observed pattern of SNP density along the 11 homeologous chromosome groups in *C. arabica* is consistent with its assumed allopolyploid structure. Furthermore, large genomic duplications or deletions were not detected, confirming low chromosomal divergence between genomes E and C of the two diploid progenitor species (Pinto-Maglio 2006), and suggesting that, following the original hybridization event, overall genome organization is stable in *C. arabica*.

### Evidence for homeologous exchanges

Homeologous exchanges appear to have played an important role in the evolution of the *C. arabica* genome. Indeed, regions exhibiting homeologous SNP deficit (HSD) and 4× copy number were identified across the genome. These regions, which ranged from 50 kb to 1170 kb in size, suggest major genetic changes. Although certainly underestimated, since the method used here cannot resolve small regions (a minimum of 50 kb was required), these regions represented nearly 1.5% of the portion of *C. arabica* genome analyzed. Furthermore, among the 9047 duplicate homeologous genes whose fate was successfully inferred in the two accessions of *C. arabica* analyzed, 2.0% were found to be affected by genomic changes leading to homeolog loss without sequence deletion in most cases sequence. Two mechanisms have been suggested to lead to such exchanges of homeologous chromatids: via crossovers and the subsequent segregation of one parental and one recombinant chromatid, or via noncrossover exchange, also called gene conversion (Gaeta and Pires 2010). Due to their large size, homeologous crossover exchanges are the most likely mechanism at the origin of the *C. arabica* regions exhibiting HSD. In contrast, both mechanisms are hypothesized to be involved in the occurrence of genes exhibiting homeolog losses. While the regions carrying contiguous genes exhibiting homeolog losses correspond to regions exhibiting HSD, most single homeolog loss events likely result from gene conversion. The high frequency of homeologous contact across the 11 homeologous chromosome groups is a surprising result given the apparently complete bivalent formation at meiosis. However, meiotic abnormalities have been repeatedly observed in *C. arabica* (Grassias and Kammacher 1975; Owuor 1985). Although exceptional, weakness in the control of the chromosome pairing in *C. arabica* could therefore enable homeologous exchanges. A possibility is that most of these homeologous exchanges, having occurred early, could be the result of possible multivalent (or less strict bivalent) pairing at meiosis at inception. Similar high frequencies of noncrossover homeologous exchanges have been reported in other genome-wide analyzed allotetraploids such as *Gossypium hirsutum* and *Brassica napus* (Salmon *et al.* 2010; Guo *et al.* 2014; Chalhoub *et al.* 2014), suggesting an important mechanism by which allopolyploid genomes adapt to the duplicate state.

Furthermore, the two main homeologous crossover exchanges detected in the two sequenced accessions of *C. arabica* were also evidenced in all analyzed accessions (*i.e.*, 96 accessions) originating from the main regions of *C. arabica* primary center of diversity. This observation strongly support the hypothesis of a single origin for *C. arabica* (Lashermes *et al.* 2014). All *C. arabica* populations would derive from a unique allopolyploidization event associated with large and specific homeologous crossover exchanges.

### Homeolog silencing suggests epigenetic changes

Gene silencing is a common response to polyploidy and has been described in many allopolyploids, including *Arabidopsis* (Wang *et al.* 2006), cotton (Chaudhary *et al.* 2009), *Tragopogon* (Buggs *et al.* 2011), and wheat (Bottley *et al.* 2006). Silencing can occur as early as the first generation following polyploidy, and some duplicated genes may be silenced in some organs of the plant but continue to be expressed in other organs (Adams and Wendel 2005). By combining RNA-seq and DNA-seq data, homeolog silencing was inferred for 1.7% of genes in the two accessions of *C. arabica* analyzed in the present study. Since gene expression was estimated only in leaves, partition of the expression of duplicate genes to specialized tissue-specific expression activity was not investigated. Silencing mechanisms almost certainly vary with the gene. In the absence of sufficient time for point mutations to accumulate in promoter regions or other *cis*-regulatory elements, most gene silencing is believed to be induced epigenetically (Adams and Wendel 2005). Intergenomic interactions between progenitor genomes in allopolyploids are predicted to induce epigenetic changes, including histone modifications and DNA methylation (Song and Chen 2015). In the last few years, gene silencing via epigenetic changes has been documented in several plants including *Tragopogon* (Sehrish *et al.* 2014), *Arabidopsis* (Chen *et al.* 2008), and *Brassica rapa* (Cheng *et al.* 2016).

### Two temporally distinct phases of evolution

The implication of homeolog loss and silencing events in the establishment and diversification of *C. arabica* is questionable (Madlung and Wendel 2013). Comparison of the two analyzed accessions revealed the occurrence of shared genomic changes, whereas other events appeared specific to one accession. While 80% of genes exhibiting homeolog loss were shared by the two accessions, only half of genes exhibiting homeolog silencing seemed shared by the two accessions. In particular, the four detected genomic regions exhibiting HSD and contiguous homeolog losses were shared by the two accessions. Two temporally distinct phases of evolution can therefore be hypothesized. A first phase accompanying the allopolyploidization process, and involving mainly homeologous crossover exchanges, could have been followed by a more gradual phase of duplicate gene evolution involving gene conversion and homeolog silencing.

Furthermore, patterns of gene loss and retention could be explained by the gene-balance hypothesis (Freeling 2009; Feldman *et al.* 2012). Under this hypothesis, the evolution of genes is linked to their function within networks (Edger and Pires 2009), so genes coding for products that are closely connected would be dosage-sensitive genes, and are thought to be retained or eliminated together to preserve stoichiometry. This evolution mechanism was recently proposed for homeologs in *T. miscellus*, a young natural allopolyploid species (Buggs *et al.* 2012). This process is probably marginal in the pattern of gene loss observed in C. *arabica* given the lack of significant GO term enrichment in the subset of genes showing homeolog loss. However, an equivalent trend might play a role in the gene silencing pattern of *C. arabica*. Indeed, genes related to macromolecular biosynthesis and organic cyclic compound metabolism tended to be preserved from gene silencing, suggesting gene dosage requirements (Veitia *et al.* 2013).

### Asymmetric evolution

Studies on duplicated genomes or genomic regions in ancient polyploids showed that they have often experienced unequal gene losses (or genome fractionation), with one genome or genomic region retaining more genes (dominant) than the other (more fractionated). More interestingly, genes located on the dominant genome or genomic region tend to have higher expression levels (Schnable *et al.* 2011; Cheng *et al.* 2012; Garsmeur *et al.* 2014; Li *et al.* 2014). Such genome dominance phenomena have been reported in a few plants including *Arabidopsis thaliana* (Wang *et al.* 2006), *Brassica* (Cheng *et al.* 2012), maize (Schnable *et al.* 2011), and wheat (Li *et al.* 2014; Wang *et al.* 2016). In *C. arabica*, homeolog loss and silencing were attributed to both subgenomes, with a marked preference for subgenome C^a^, suggesting dominance of the E^a^ genome. However, neither of the two subgenomes appeared to be preferentially expressed in *C. arabica* (Combes *et al.* 2013), or in interspecific hybrids between *C. canephora* and *C. eugenioides* (Combes *et al.* 2015). The absence of relationships between the observed preferential changes in the C^a^ genome and overall homeologous expression could be linked to the recent origin and the intertwined homeolog regulations occurring in *C. arabica* (Combes *et al.* 2013).

### Conclusions

The present results point to overall structural genome stability in *C. arabica* following the original hybridization event. However, homeologous DNA exchanges and homeolog silencing were evidenced that could have played a major role in the stabilization and survival of the ancestral allotetraploid, and in its subsequent diversification. While the early phase of evolution involved mainly homeologous crossover exchanges, the later phase appears to have relied on a more gradual phase of duplicate gene evolution involving gene conversion and homeolog silencing. As suggested in *C. arabica*, the evolutionary success of a newly formed polyploidy may require a delicate balance between genetic and epigenetic changes.

## Supplementary Material

Supplemental Material
