# Supplementary material for: Inter-genomic DNA Exchanges and Homeologous Gene Silencing Shaped the Nascent Allopolyploid Coffee Genome (Coffea arabica L.)
Source: G3 (Bethesda). 2016 Jul 19;6(9):2937–48. doi: 10.1534/g3.116.030858 (PMC5015950; doi:10.1534/g3.116.030858)
Supplement: Supplemental Material [file supp_6_9_2937__index.html]

Inter-genomic DNA Exchanges and Homeologous Gene Silencing Shaped the Nascent Allopolyploid Coffee Genome (Coffea arabica L.) — Supplemental Material 

# Inter-genomic DNA Exchanges and Homeologous Gene Silencing Shaped the Nascent Allopolyploid Coffee Genome (*Coffea arabica* L.)

## Supplemental Material for Lashermes *et al.*, 2016

**Files in this Data Supplement:**

- Figure S1 - Read depth measurements of genes of *C. arabica* (acc. Caturra) (.pdf, 191 KB)
- Table S1 - Total number of reads sequenced and mapped onto the *C. canephora* (acc. DH200-94) reference genome after sequencing of two accessions of *C. arabica*. (.xlsx, 10 KB)
- Table S2 - Identification of regions exhibiting putative homoeologous SNP deficit (HSD) in *C. arabica* (acc. Caturra). (.xlsx, 3 MB)
- Table S3 - Gene ontology enrichment analysis of genes exhibiting homoeolog silencing in *C. arabica*. (.xlsx, 10 KB)
- Table S4 - Genes investigated, pairs of oligonucleotides and SNP used to validate genes exhibiting homoeolog lost or silencing in *C. Arabica*. (.xlsx, 12 KB)
- Table S5 - List of *C. arabica* accessions used to validate the presence of genomic regions exhibiting homoeologous SNP deficit and to analyze their distribution among the *C. arabica* germplasm. (.xlsx, 13 KB)
