# Supplementary material for: Inter-genomic DNA Exchanges and Homeologous Gene Silencing Shaped the Nascent Allopolyploid Coffee Genome (Coffea arabica L.)
Source: G3 (Bethesda). 2016 Jul 19;6(9):2937–48. doi: 10.1534/g3.116.030858 (PMC5015950; doi:10.1534/g3.116.030858)

Figure S1. Read depth measurements of genes of *C. arabica* (acc. Caturra). Distribution is shown according to the average read depth of coverage of all genes, genes exhibiting homoeolog loss or silencing. Only the set of 9,047 genes analyzed in the two accessions was taken into consideration.

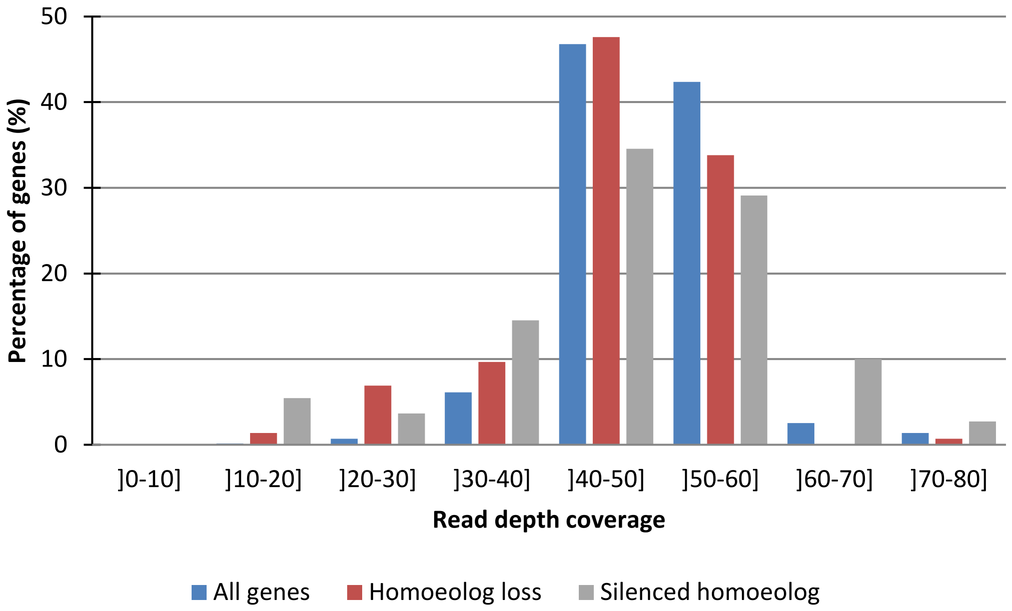

Supplement: Supplemental Material [file supp_g3.116.030858_FigureS1.pdf]
